# Indices of leg resistance artery function are independently related to cycling V̇O_2_max

**DOI:** 10.14814/phy2.14551

**Published:** 2020-08-18

**Authors:** Jayson R. Gifford, Brady E. Hanson, Meagan Proffit, Taysom Wallace, Jason Kofoed, Garrett Griffin, Melina Hanson

**Affiliations:** ^1^ Department of Exercise Sciences Brigham Young University Provo UT USA; ^2^ Program of Gerontology Brigham Young University Provo UT USA

**Keywords:** flow‐mediated dilation, passive leg movement, rapid onset vasodilation, vascular function, V̇O_2_max

## Abstract

**Purpose:**

While maximum blood flow influences one's maximum rate of oxygen consumption (V̇O_2_max), with so many indices of vascular function, it is still unclear if vascular function is related to V̇O_2_max in healthy, young adults. The purpose of this study was to determine if several common vascular tests of conduit artery and resistance artery function provide similar information about vascular function and the relationship between vascular function and V̇O_2_max.

**Methods:**

Twenty‐two healthy adults completed multiple assessments of leg vascular function, including flow‐mediated dilation (FMD), reactive hyperemia (RH), passive leg movement (PLM), and rapid onset vasodilation (ROV). V̇O_2_max was assessed with a graded exercise test on a cycle ergometer.

**Results:**

Indices associated with resistance artery function (e.g., peak flow during RH, PLM, and ROV) were generally related to each other (*r* = 0.47–77, *p* < .05), while indices derived from FMD were unrelated to other tests (*p* < .05). Absolute V̇O_2_max (*r* = 0.57–0.73, *p* < .05) and mass‐specific V̇O_2_max (*r* = 0.41–0.46, *p* < .05) were related to indices of resistance artery function, even when controlling for factors like body mass and sex. FMD was only related to mass‐specific V̇O_2_max after statistically controlling for baseline artery diameter (*r* = 0.44, *p* < .05).

**Conclusion:**

Indices of leg resistance artery function (e.g., peak flow during RH, PLM, and ROV) relate well to each other and account for ~30% of the variance in V̇O_2_max not accounted for by other factors, like body mass and sex. Vascular interventions should focus on improving indices of resistance artery function, not conduit artery function, when seeking to improve exercise capacity.

## Graphical abstract

Indices of resistance artery function, but not conduit artery function, are strongly related to exercise capacity assessed by V̇O_2_max during cycling.

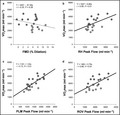

## 1 INTRODUCTION

One's maximum rate of oxygen consumption (V̇O_2_max) strongly influences exercise performance and is also a strong predictor of cardiovascular risk (Poole, Behnke, & Musch, 2020). While many systems may limit V̇O_2_max (Wagner, 2008), the cardiovascular system often serves as a significant bottleneck, with untrained individuals often exhibiting a much lower cardiac output and muscle blood flow than endurance‐trained individuals (Gifford et al., 2016; Levine, 2008). Evidence indicates that cardiac output and muscle blood flow during exercise are both strongly influenced by the ability of the peripheral vasculature to dilate (Bada, Svendsen, Secher, Saltin, & Mortensen, 2012; Hanson, Proffit, & Gifford, 2020; Joyner & Casey, 2015). While large conduit arteries, like the brachial or femoral arteries, may dilate during exercise (Tremblay & Pyke, 2018), it is the dilation of the network of small resistance arteries, whose total cross‐sectional area far exceeds that of the large conduit arteries (Wiedman, 1963), that primarily regulates the increase in blood flow during exercise (Joyner & Casey, 2015; VanTeeffelen & Segal, 2006).

With fluctuations in the radius of the arterial circulation having such a profound impact on blood flow and cardiac output, several studies have sought to determine if the ability of the vasculature to dilate, often termed vascular function (Gifford & Richardson, 2017), is related to exercise capacity (Montero, 2015).

In such studies vascular function has usually (Montero, 2015) been quantified with a technique that measures the vasodilator ability of a conduit artery (Flow‐Mediated Dilation, FMD) in a region not majorly involved in most tests of V̇O_2_max, the arm. Despite vascular function being measured in a conduit artery that does not perfuse the main exercising muscles, most studies demonstrate a significant, positive relationship between brachial FMD and V̇O_2_max during running or cycling exercise (Montero, 2015). However, as noted by Montero (Montero, 2015), the comparison of conduit artery function of an upper limb to the V̇O_2_max elicited by a lower‐body exercise (e.g., cycling or running) is problematic since upper‐limb vascular function is not reflective of lower‐limb vascular function (Thijssen, Rowley, et al., 2011), and exercise training is known to elicit local adaptations in conduit artery structure that may mask any adaptation in local vascular function (Green, Spence, Rowley, Thijssen, & Naylor, 2012). Moreover, given the negligible role of conduit arteries in regulating exercise blood flow (Joyner & Casey, 2015), the relevance of conduit artery function to exercise capacity is unclear.

Multiple noninvasive assessments intended to interrogate the function of the resistance arteries (Limberg et al., 2020) of the lower limbs have been developed in recent years. Indeed, tests such as reactive hyperemia (RH) in response to cuff occlusion and removal, the hyperemic response to passive leg movement (PLM), and the rapid onset of vasodilation and hyperemia in response to a single muscle contraction (ROV) have been shown to be NO dependent (Broxterman et al., 2017; Casey, Walker, Ranadive, Taylor, & Joyner, 2013; Gifford & Richardson, 2017; Limberg et al., 2020) and related to peak blood flow during knee extension exercise (Hanson et al., 2020). Nevertheless, it is not clear if these distinct indices of vascular function actually reflect the same underlying physiology and are related to each other. It is also unclear how relevant these indices of resistance artery function (Limberg et al., 2020) are to exercise capacity. If these indices of resistance artery vascular function truly are representative of the function of the resistance arteries, which are largely responsible for blood flow control (Joyner & Casey, 2015), one may expect them to relate well to exercise capacity.

To date, the relationships between the various tests of vascular function in the lower limb and their relevance to exercise capacity have not been extensively explored. Therefore, the purpose of this study was twofold. First, we sought to determine how well the various indices of conduit and resistance artery function relate to each other. Second, we sought to determine if aerobic exercise capacity, assessed by V̇O_2_max during cycling exercise, is related to various indices of conduit artery and resistance artery function.

## 2 METHODS

### 2.1 Subjects

Twenty‐two young, healthy subjects (13 males, 9 females, 18–30 years old) completed the study. All subjects were healthy, nonobese, nonsmokers, free from medications that would affect their hemodynamic responses to exercise (Gifford & Richardson, 2017). Data for females were collected within the first 7 days of the menstrual cycle to minimize variability attributable to hormonal fluctuations. Prior to starting the study, the Institutional Review Board (IRB) at Brigham Young University (BYU) found the study to be safe, ethical, and in agreement with the main principles outlined in the Declaration of Helsinki. Prior to participation, all subjects provided informed consent. While the study was performed in accordance principles outlined in the *Declaration of Helsinki,* it was not registered on clinicaltrials.gov before data collection.

### 2.2 Procedures

Subjects reported to the laboratory on three occasions having fasted for 4 hr, rested from exercise and refrained from alcohol or caffeine consumption for ~24 hr (Gifford & Richardson, 2017). Each visit was separated by a minimum of 24 hr. All data collection was completed on the subject's right leg, regardless of leg dominance.

On the first visit, body measurements including height (cm), body mass (kg), and body mass index (BMI, kg·m^−2^). After resting supine for 20 min, vascular function was assessed by the FMD and RH techniques on the superficial femoral artery as described below. Subsequently, the maximum rate of oxygen consumption during cycling (i.e., V̇O_2_max) was assessed with a graded exercise test (25 watt increments per minute) on a cycle ergometer (Excaliber Sport, Lode, Groningen, Netherlands) with a Parvo metabolic cart (True One, Parvo‐Medics Inc., Sandy, Utah, USA) (Gifford et al., 2016)). The greatest power sustained for 1 min during the graded exercise test was identified as Graded Exercise Test Max (GXTmax). Following 30 min of rest from the initial graded exercise test, a constant‐load test (100% GXTmax) until exhaustion test was performed to verify the initial V̇O_2_max results (Poole & Jones, 2017). The verification V̇O_2_max for all subjects was within ±5% of the initial V̇O_2_max, supporting the attainment of V̇O_2_max. The higher of the two values was recorded as the final V̇O_2_max.

On the second visit, vascular function was assessed first with the passive leg movement (PLM) technique in triplicate. Following a ~ 5‐min recovery period, vascular function was then assessed by the rapid onset vasodilation (ROV) technique elicited by single kick knee extension exercise as described below.

### 2.3 Assessments of vascular function

#### 2.3.1 Flow‐mediated dilation (FMD) and reactive hyperemia (RH)

During the first visit subjects reported to the laboratory to have vascular function assessed via FMD and RH on the superficial femoral artery according to current recommendations (Harris, Nishiyama, Wray, & Richardson, 2010) and as previously described (Hanson et al., 2020). While lying in the supine position, a 9 cm blood pressure cuff (Hokanson Inc., Bellevue, WA, USA) was placed on the thigh proximal to the kneecap. Following a 20‐min acclimation/resting period, baseline measurements (diameter and blood flow) were gathered for 60 s at the superficial femoral artery ~10 cm proximal to the cuff with a GE Logiq E ultrasound (General Electric Medical Systems, Milwaukee, WI, USA) operating with a B‐mode frequency of 9 MHz and a Doppler frequency of 5 MHz. The cuff was then inflated for 5 min to 250 mmHg. Blood velocity and diameter data were collected for 2 min immediately after the release of the cuff pressure. Following the study, artery diameter was analyzed frame‐by‐frame by automated edge detection software (Quipu srl., Pisa, Italy) and averaged into 1‐s bins corresponding to 1‐s average velocities. A 3‐s rolling average was applied to smooth diameter and velocity data. Blood flow (ml·min^−1^) was calculated using the equation: $\text{blood flow}=\left[\left(\text{mean blood velocity}\right)\times (\pi \times ({\text{vessel radius}}^{2}\right))\;x\;60]$, where mean blood velocity is expressed in cm·s^−1^ and radius is expressed in cm. FMD measurements were expressed as a percent change in diameter and calculated with the equation:

$$\text{FMD}\left(\%\right)=\frac{\left(\text{Peak Diameter}‐\text{Baseline Diameter}\right)}{\text{Baseline Diameter}}\times 100.$$

Shear rate was calculated with the following equation $\text{Shear rate}=\frac{8\times \text{mean blood velocity}}{\text{Diameter}}$. Subsequently FMD was also normalized for total shear area under the curve (i.e., FMD/shear) as recommended and described by Harris et al (Harris et al., 2010). Peak flow during RH following the release of the cuff was identified as the greatest 1‐s average of flow achieved following cuff release (Harris et al., 2010).

#### 2.3.2 Passive leg movement (PLM)

The hyperemic response induced by PLM, which is NO‐dependent (Broxterman et al., 2017; Mortensen, Askew, Walker, Nyberg, & Hellsten, 2012; Trinity et al., 2012) and strongly related to acetylcholine‐induced hyperemia (Mortensen et al., 2012), was utilized to assess thigh vascular function according to recently published guidelines (Gifford & Richardson, 2017). Subjects were seated in an upright position with knees fully extended (180°) for a 20‐min acclimation period before any data were collected. Subsequently, resting blood flow was measured for 60 s at the common femoral artery utilizing a GE Logiq E ultrasound (General Electric Medical Systems, Milwaukee, WI, USA) operating with a B‐mode frequency of 9 MHz, a Doppler frequency of 5 MHz, and an insonation angle of 60°. Subsequently, researchers manually moved the subject's leg back and forth from the extended position of the knee (180°) to the flexed position (90°), at a rate of 60 knee extensions per min, while the subjects stayed relaxed with no voluntary muscle contraction, while blood flow was measured at the common femoral artery throughout. This procedure was completed three times with a ~15‐min period of rest between each trial. Blood flow data were analyzed second‐by‐second and a 3‐s rolling average was applied to smooth the data. The peak blood flow and the area under the curve (PLM Total Flow) were identified for each of the three trials and then averaged together (Gifford & Richardson, 2017). The data presented in this manuscript are the average of the three trials.

#### 2.3.3 Rapid onset vasodilation (ROV)

The hyperemic response to a single muscle contraction (e.g., one leg extension) has also been shown to be NO dependent (Casey et al., 2013) and is indicative of the responsiveness of the vasculature to an exercise stimulus (Credeur et al., 2015; Hughes, Ueda, & Casey, 2016). For this study, the hyperemic response to a single knee extension of 60 Nm of work was used to quantify ROV. Subjects were seated in an upright position with legs hanging over the end of a seat with knees in a flexed position (knee at 90° flexion at rest). The right ankle was then connected to the cable of a knee extension machine (a basic pulley system that vertically displaces a selected amount of weight – N.K. Products, Lake Elsinore, CA, USA). Subjects then fully extended their leg so that the vertical displacement distance associated with a fully extended kick could be measured using a standard tape measure. This displacement distance was subsequently used to calculate the total work performed during the different kicks. Subjects were then familiarized with the kicking motion at various different weights.

Following 20‐min recovery, ROV was assessed in duplicate in response to a full knee extension totaling 60 Nm of work. Repeated trials were separated by at least 2 min of recovery. As subjects of different leg lengths displaced the weights to different distances, the mass each subject lifted was adjusted for the absolute work kick so that the total work (i.e., Total Work = mass × gravity × displacement distance) was 60 Nm when extending the leg through a full 90° range of motion. For each kick, 1 min of baseline data was collected while the leg was rested in a flexed position. Subsequently, subjects extended the knee to ~180° and then passively allowed the weight to flex the knee back to 90° with no engagement of knee flexor or extensor muscles during the knee flexion phase (e.g., active contraction during knee extension and no contraction during flexion). Femoral blood flow was assessed, as described for the PLM technique, for 1 min of baseline prior to contraction, during the kick and for 1 min following the kick. Data were subsequently analyzed second‐by‐second and a 3‐s rolling average was applied to smooth the data. The peak blood flow (ROV Peak Flow) was subsequently identified as the greatest 1‐s average of blood flow, while the total flow response (ROV Total Flow) was identified as the area under the curve for 60 s. As each exercise was performed in duplicate, the data reported in this manuscript are the average of both trials.

### 2.4 Statistical analysis

Test–retest reliability of the variables that were performed in repeated measures (PLM‐based indices in triplicate and ROV‐based indices in duplicate) was assessed with intraclass correlation (ICC) using a two‐way mixed model based on absolute agreement. As the average of the multiple measurements was used for the analysis in this study, the ICC for the average of the repeated measures, not the ICC for an individual measure, is reported. Criteria for classifying the level of reliability of measurements were based up those set forth by Koo & Li (2016), in which an ICC between 0.50 and 0.75 is evidence of “moderate reliability”, an ICC between 0.75 and 0.90 is evidence of “good reliability”, and an ICC > 0.90 is evidence of “excellent reliability”.

Pearson correlation and a linear regression were utilized to determine the relationship between the various assessments of vascular function and other variables. Categorical data, like sex, were dummy coded into correlations. Part/partial correlation was utilized to determine the amount of unique variance shared by two variables when removing that related to a third variable. Principal components analysis was utilized to combine the large amount of information provided by the multiple indices of vascular function into fewer, discrete variables based on the shared variance among the different indices of vascular function. Specifically, major variables derived from the tests of vascular function (FMD % dilation, FMD/shear, RH Peak Flow, RH Total Flow, PLM Peak Flow, PLM Total Flow, ROV Peak Flow, and ROV Total Flow) were entered into a principal components analysis with orthogonal rotation (varimax). Factors with an eigenvalues greater than 0.7 were accepted and only variables with loadings greater than 0.7 were included in a factor (Field, 2009). An independent sample *t*‐test was conducted to identify sex differences among the indices of vascular function. Alpha was set at *p* ≤ .05 a priori.

All statistical analyses were completed using SPSS version 26 (SPSS Inc.). Data are expressed as the mean ± SE unless otherwise stated.

## 3 RESULTS

### 3.1 Test–retest reliability of PLM and ROV measurements

The repeated measurements of PLM Peak Flow and PLM Total Flow both exhibited “excellent reliability” with ICC equal to 0.91. The repeated measurements of ROV Peak Flow exhibited “excellent reliability” with ICC equal to 0.96. The repeated measurements of ROV Total Flow exhibited “moderate reliability” with ICC equal to 0.72. As mentioned in the methods section, the average of the repeated measurements was utilized for all subsequent analyses in this study.

### 3.2 Relationship between the various assessments of vascular function

**Figure 1:**
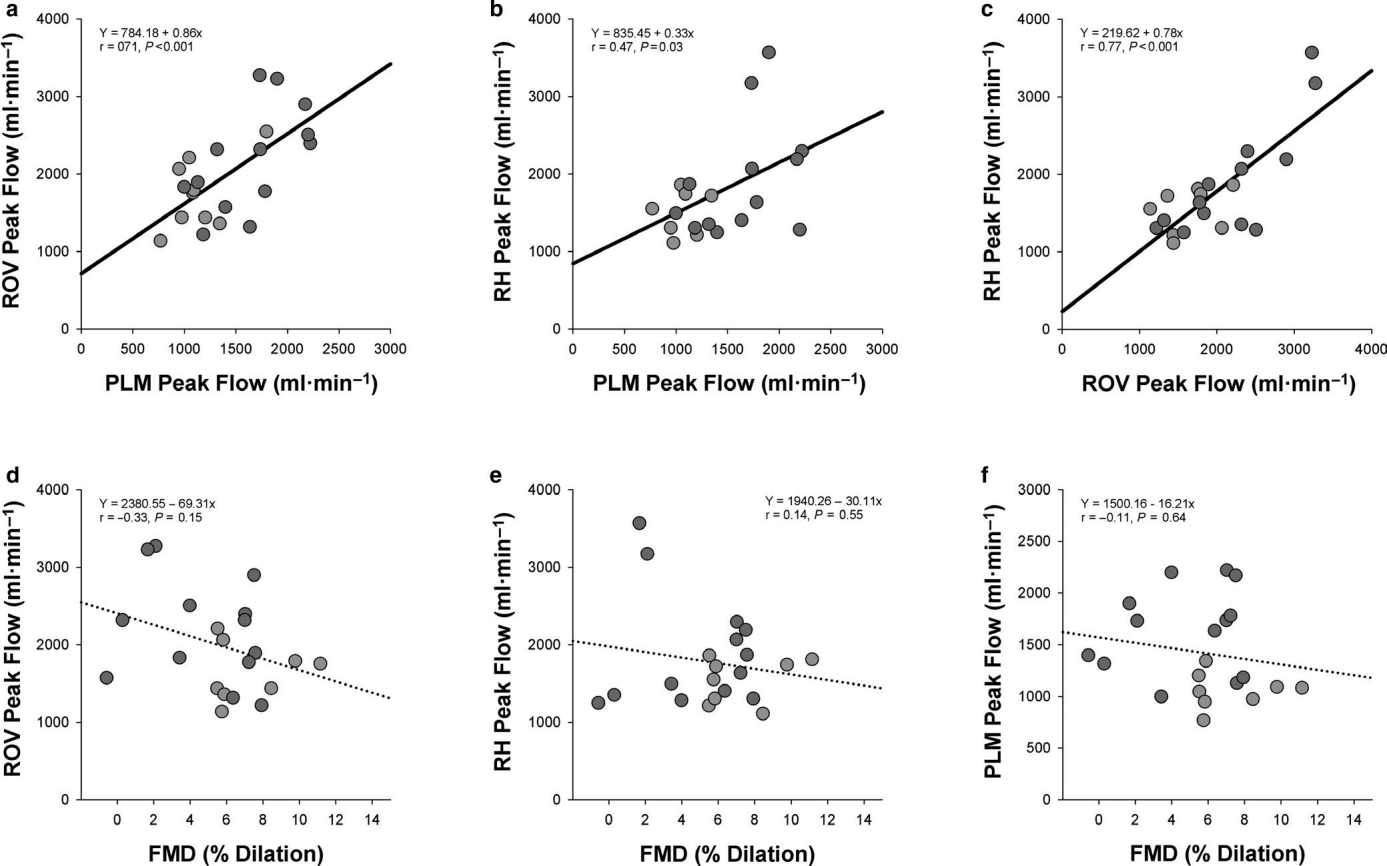
Relationship between Different Indices of Vascular Function. (a) Relationship between the peak flow achieved during passive leg movement (PLM) and the peak flow achieved during the rapid onset vasodilation (ROV) test. (b) Relationship between PLM peak flow and the peak flow observed during a reactive hyperemia (RH) test. (c) Relationship between the peak flow achieved during the ROV and RH tests. (d) Relationship between flow‐mediated dilation (FMD) of the superficial femoral artery and the peak flow achieved during an ROV test. (e) Relationship between FMD of the superficial femoral artery and the peak flow achieved during RH. (f) Relationship between FMD of the superficial femoral artery and the peak flow achieved during PLM. A solid trendline indicates a significant relationship between the two variables (*p ≤ *.05) while a dotted trendline indicates a nonsignificant relationship between the two variables (*p* > .05). Light gray circles represent data for females and dark gray circles represent data for males

As illustrated in Figure 1 and further described in Table 1, the relationships between the multiple indices of vascular function were examined with Pearson correlation. In general, indices derived from resistance artery function tests (i.e., RH, PLM, and ROV) were related to each other (*p* < .05), but not to indices derived from conduit artery function tests (e.g., FMD, *p* > .05).

**Table 1:** Relationship between various indices of vascular function

					FMD (% Dilation)		FMD (%/ Shear)		RH peak flow (ml/min)		RH total flow (ml)		PLM peak flow (ml/min)		PLM total flow (ml)		ROV peak flow (ml/min)		ROV total flow (ml)	
Factor 1		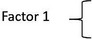		FMD (% Dilation)		‐		***r* = 0.47** ***p* = .04**		*r* = −0.14 *p* = .55		*r* = −0.01 *p* = .99		*r* = −0.11 *p* = .64		*r* = 0.01 *p* = .96		*r* = −0.33 *p* = .15		*r* = −0.04 *p* = .85
				FMD (%/ Shear)		***r* = 0.47** ***p* = .04**		‐		*r* = −0.21 *p* = .37		***r* = −0.54** ***p* = .01**		*r* = −0.31 *p* = .18		*r* = −0.07 *p* = .78		*r* = −0.32 *p* = .17		*r* = 0.18 *p* = .45
Factor 2		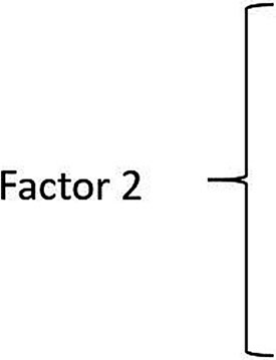		RH peak flow (ml/min)		*r* = −0.14 *p* = .55		*r* = −0.21 *p* = .37		‐		***r* = 0.82** ***p* < .01**		***r* = 0.47** ***p* = .03**		*r* = 0.31 *p* = .17		***r* = 0.77** ***p* < .01**		*r* = 0.36 *p* = .11
				RH total flow (ml)		*r* = −0.01 *p* = .99		***r* = −0.54** ***p* = .01**		***r* = 0.82** ***p* < .01**		‐		***r* = 0.40** ***p* = .08**		*r* = 0.23 *p* = .32		***r* = 0.57** ***p* = .01**		*r* = 0.09 *p* = .71
				PLM peak flow (ml/min)		*r* = −0.11 *p* = .64		*r* = −0.31 *p* = .18		***r* = 0.47** ***p* = .03**		***r* = 0.40** ***p* = .08**		‐		***r* = 0.89** ***p* < .01**		***r* = 0.64** ***p* < .01**		*r* = 0.24 *p* = .28
				PLM total flow (ml)		*r* = 0.01 *p* = .96		*r* = −0.07 *p* = .78		*r* = 0.31 *p* = .17		*r* = 0.23 *p* = .32		***r* = 0.89** ***p* = .01**		‐		***r* = 0.45** ***p* = .04**		*r* = 0.18 *p* = .41
				ROV peak flow (ml/min)		*r* = −0.33 *p* = .15		*r* = −0.32 *p* = .17		***r* = 0.77** ***p* < .01**		***r* = 0.57** ***p* = .01**		***r* = 0.64** ***p* < .01**		***r* = 0.45** ***p* = .04**		‐		***r* = 0.63** ***p* < .01**
				ROV total flow (ml)		*r* = −0.04 *p* = .85		*r* = 0.18 *p* = .45		*r* = 0.36 *p* = .11		*r* = 0.09 *p* = .71 .01		*r* = 0.24 *p* = .28		*r* = 0.18 *p* = .41		***r* = 0.63** ***p* < .01**		‐

Note

The terms “Factor 1” and “Factor 2” at the left of the table refer to the variables that were grouped together via principal components analysis. Significant relationships are in bold font

Abbreviations: FMD, flow‐mediated dilation; PLM: passive leg movement; RH: Reactive Hyperemia; ROV: Rapid onset vasodilation.

Principal components analysis of the variables listed in Table 1 was utilized to group indices that share substantial variance to condense the multiple indices of vascular function to fewer factors. In essence, this analysis determines the extent to which the various assessments of vascular function represent similar or distinct factors. The Kaiser‐Meyer‐Olkin measure (KMO = 0.54) supported the sampling adequacy for the factor analysis. Visual analysis of a scree plot indicated a breakpoint at two factors, supporting the inclusion of two different factors with eigenvalues greater than 0.7. Factor #1 was exclusively comprised of factors related to FMD (FMD % dilation and FMD/shear) with loading factors of 0.72 and 0.89, respectively. Factor #2 was comprised of the following variables with the loading factors indicated in parentheses: RH Peak Flow (0.84), RH Total Flow (0.75), PLM Peak Flow (0.83), PLM Total Flow (0.74), and ROV Peak Flow (0.84). ROV total flow was not included in either factor.

### 3.3 Relationship between indices of vascular function and V̇O_2_max

**Figure 2:**
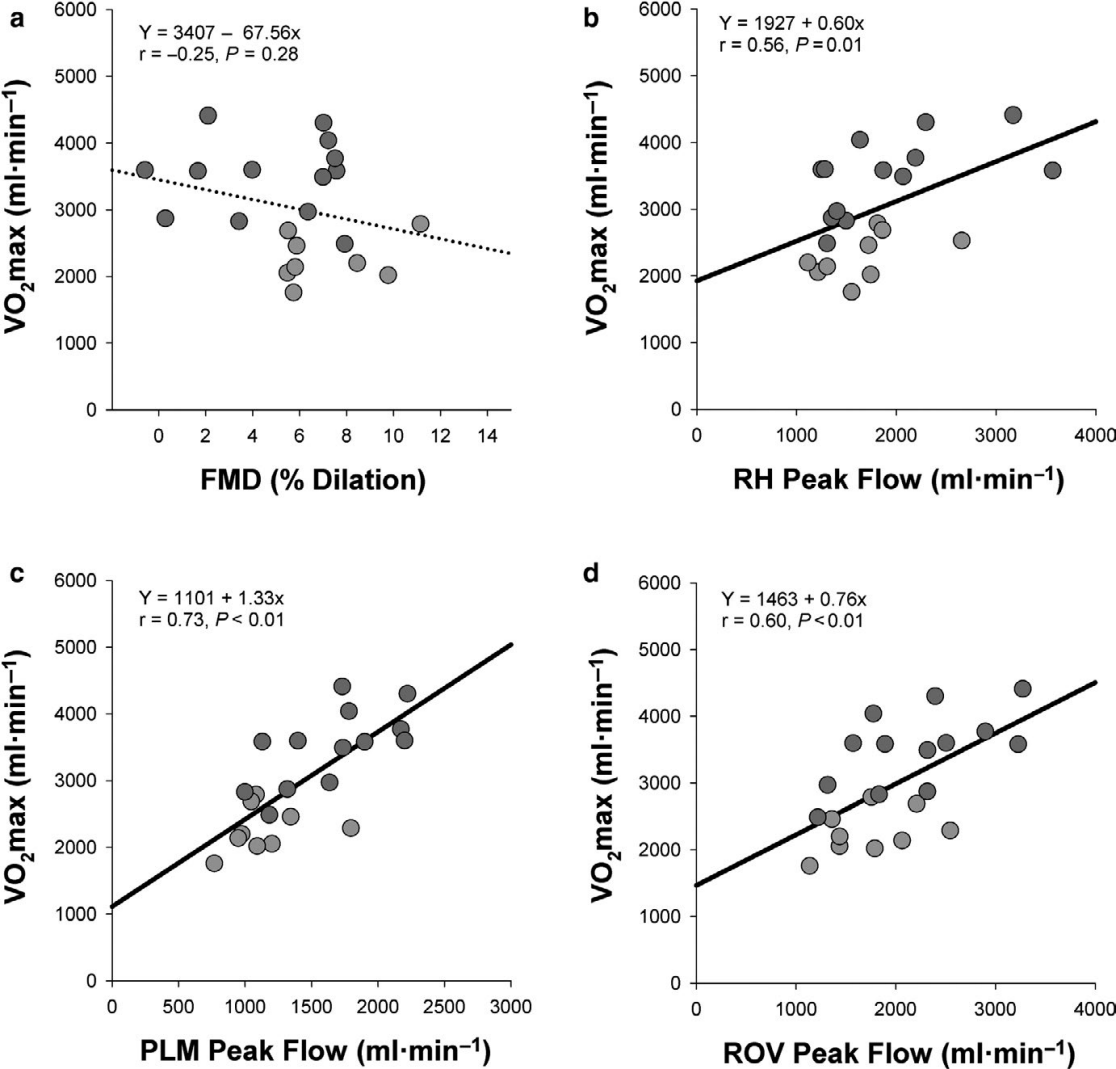
The Relationship between Vascular Function and The Maximum Rate of Oxygen Consumption (V̇O_2_max) during Cycling. The relationship between absolute V̇O_2_max and (a) flow‐mediated dilation (FMD) of the superficial femoral artery (b) peak flow during Reactive hyperemia (RH), (c**)** peak flow during passive leg movement (PLM) and (d**)** peak flow during a rapid onset vasodilation (ROV) test. V̇O_2_max. A solid trendline indicates a significant relationship between the two variables (*p ≤ *.05), while a dotted trendline indicates a nonsignificant relationship between the two variables (*p* > .05). Light gray circles represent data for females and dark gray circles represent data for males

As illustrated in Figure 2 and further described in Table 2, variables associated with FMD were unrelated to mass‐specific and absolute V̇O_2_max (*p* = .12–0.40). Meanwhile, variables associated with the second factor revealed in factor analysis (e.g., RH Peak Flow, PLM Peak Flow, and ROV Peak Flow) exhibited moderate‐to‐strong correlations with absolute and mass‐specific V̇O_2_max (*r* = 0.56–73, *p* < .05).

**Table 2:** Relationship between different indices of vascular function and maximum rate of oxygen consumption (V̇O_2_max) achieved during cycling

	Mass‐Specific V̇O_2_max (ml/kg/min)	Absolute V̇O_2_max (ml/min)
FMD (% Dilation)	*r* = 0.20 *p* = .40	*r* = −0.24 *p* = .31
FMD (%/shear)	*r* = −0.27 *p* = .24	*r* = −0.35 *p* = .12
RH Peak Flow (ml/min)	*r* = 0.26 *p* = .25	***r* = 0.49** ***p* = .02**
RH Total Flow (ml)	*r* = 0.41 *p* = .06	***r* = 0.44** ***p* = .04**
PLM Peak Flow (ml/min)	***r* = 0.48** ***p* = .03**	***r* = 0.72** ***p* < .01**
PLM Total Flow (ml)	***r* = 0.42** ***p* = .05**	***r* = 0.65** ***p* < .01**
ROV Peak Flow (ml/min)	*r* = 0.36 *p* = .11	***r* = 0.58** ***p* < .01**
ROV Total Flow (ml)	*r* = 0.10 *p* = .65	*r* = 0.03 *p* = .91
Body Mass (kg)	*r* = 0.16 *p* = .49	***r* = 0.84** ***p* < .01**
Body Mass Index (kg/m^2^)	*r* = −0.16 *p* = .49	***r* = 0.64** ***p* < .01**
Sex (Female = −1, Male = +1)	*r* = 0.01 *p* = .98	***r* = 0.79** ***p* < .01**

Note

Note that sex has been dummy coded with females being coded as −1 and males being entered as + 1. In this dummy coding scenario, a negative correlation indicates greater values are associated with the female sex, while a positive correlation indicates greater values are associated with the male sex. Significant relationships are in bold font

Abbreviations: FMD, flow‐mediated dilation; PLM, passive leg movement; RH, reactive hyperemia; ROV, rapid onset vasodilation.

### 3.4 Other factors that relate to the indices of vascular function

As described in Table 3, factors related to a subject's anatomy, sex, and body mass were related to the outcomes of the vascular function tests. Notably, FMD exhibited a negative correlation with the artery diameter at baseline (*r* = −0.64, *p* = .002), such that individuals with larger arteries tended to exhibit lower FMD (Table 3). Meanwhile, body mass was positively related with RH Peak Flow (*r* = 0.47, *p* = .01), PLM Peak Flow (*r* = 0.62, *p* < .01), and ROV Peak Flow (*r* = 0.53, *p* = .01).

**Table 3:** Relationship between indices of vascular function and other subject characteristics

	Baseline Artery Diameter (mm)	Body Mass (kg)	BMI (kg/m^2^)	Sex (Female = −1, Male = +1)
FMD (% Dilation)	***r* = −0.64** ***p* < .01**	***r* = −0.47** ***p* = .03**	***r* = −0.48** ***p* = .03**	*r* = −0.38 *p* = .09
FMD (%/shear)	***r* = −0.49** ***p* = .03**	*r* = −0.27 *p* = .26	*r* = −0.33 *p* = .17	*r* = −0.15 *p* = .54
RH Peak Flow (ml/min)	***r* = 0.65** ***p* < .01**	***r* = −0.47** ***p* = .03**	***r* = 0.48** ***p* = .03**	*r* = 0.30 *p* = .19
RH Total Flow (ml)	***r* = 0.48** ***p* = .03**	*r* = 0.28 *p* = .22	*r* = 0.31 *p* = .17	*r* = 0.17 *p* = .46
PLM Peak Flow (ml/min)	***r* = 0.85** ***p* < .01**	***r* = 0.62** ***p* < .01**	***r* = 0.59** ***p* < .01**	***r* = 0.56** ***p* < .01**
PLM Total Flow (ml)	***r* = 0.74** ***p* < .01**	***r* = 0.58** ***p* < .01**	***r* = 0.54** ***p* < .01**	***r* = 0.54** ***p* = .01**
ROV Peak Flow (ml/min)	***r* = 0.75** ***p* < .01**	***r* = 0.53** ***p* = .01**	***r* = 0.61** ***p* < .01**	*r* = 0.35 *p* = .11
ROV Total Flow (ml)	*r* = 0.18 *p* = .43	*r* = 0.01 *p* = .99	*r* = 0.15 *p* = .50	*r* = 0.03 *p* = .98

Note

Note that sex has been dummy coded with females being coded as −1 and males being entered as +1. Significant relationships are in bold font.

Abbreviations: BMI, body mass index; FMD, flow‐mediated dilation; PLM, passive leg movement; ROV, rapid onset vasodilation.

#### 3.4.1 Sex differences in indices of vascular function

PLM Peak Flow (Female: 1,140 ± 98 ml min^−1^, Male: 1626 ± 114 ml min^−1^; *p* = .006) and PLM Total Flow (Female: 333 ± 57 ml, Male: 566 ± 56 ml; *p* = .01) were both significantly greater in males than females. ROV peak flow also tended to be greater in males than females (Female: 1762 ± 149 ml min^−1^, Male: 2,196 ± 186 ml min^−1^; *p* = .10), while FMD (% Dilation) tended to be lower in males than females (Female: 6.98 ± 0.78%, Male: 4.73 ± 0.85%; *p* = .09). The sex difference in PLM Peak Flow and Total Flow disappeared when controlling for body mass (*p* = .98), which was significantly different between the females and males in the study (57.00 ± 1.69 kg vs. 82.35 ± 1.69 kg, respectively, *p* < .01).

### 3.5 Relationship between vascular function and V̇O_2_max when controlling for other variables

Recognizing that several other factors may potentially influence the responses observed in the different vascular function tests (see Table 3), the relationship between V̇O_2_max and the various indices of vascular function was examined when controlling for potentially confounding variables. When controlling for the variation in FMD accounted for by baseline diameter, FMD was found to be significantly related to the mass‐specific V̇O_2_max (*r* = 0.44, *p* = .04; Table 4). Moreover, when simultaneously accounting for the variance related to body mass, sex, and BMI with partial correlation, RH Peak Flow, PLM Peak, and ROV Peak Flow were still significantly related to absolute V̇O_2_max (*r* = 0.49–0.59, *p* < .05) and mass‐specific V̇O_2_max (*r* = 0.46–0.55, *p* ≤ .05).

**Table 4:** Partial correlations between indices of vascular function and the maximum rate of oxygen consumption (V̇O_2_max) during cycling exercise when controlling for potentially confounding variables

	Mass‐Specific V̇O_2_max (ml/kg/min)	Absolute V̇O_2_max (ml/min)
FMD (% Dilation) Controlling for baseline diameter	***r* = 0.45** ***p* = .04**	*r* = 0.35 *p* = .14
Peak Reactive Hyperemia (ml/min) Controlling for body mass, sex, and BMI	***r* = 0.46** ***p* = .05**	***r* = 0.49** ***p* = .04**
PLM Peak Flow (ml/min) Controlling for body mass, sex, and BMI	***r* = 0.53** ***p* = .02**	***r* = 0.58** ***p* = .01**
ROV Peak Flow (ml/min) Controlling for body mass, sex, and BMI	***r* = 0.55** ***p* = .01**	***r* = 0.59** ***p* < .01**

Note

Note that sex has been dummy coded with females being coded as −1 and males being entered as +1. Significant relationships are in bold font.

Abbreviations: BMI, body mass index; FMD, flow‐mediated dilation; PLM, passive leg movement; ROV, rapid onset vasodilation.

Finally, stepwise linear regression was performed to explore the possibility of predicting V̇O_2_max with vascular function data and other subject characteristics. Of the five variables entered into the regression (body mass, sex, height, BMI, and PLM Peak Flow), only body mass, PLM Peak Flow, and BMI were retained by the stepwise regression, yielding the following equation (*R* ^2^ = 0.83, *p* < .01): Absolute V̇O_2_max = 970.82 + 55.83 (Body Mass) + 0.68 (PLM Peak Flow) – 121.75 (BMI).

## 4 DISCUSSION

The purpose of this study was to determine how well the various indices of vascular function relate to each other and if aerobic capacity, assessed by V̇O_2_max, is related to these indices of vascular function. The results of this inquiry yielded two major findings. First, in agreement with current thought (Limberg et al., 2020; Thijssen, Black, et al., 2011), the assessments of conduit artery function (FMD and its derivatives) and resistance artery function (derivatives of RH, PLM, and ROV) appear to reflect two different aspects of vascular function, with the indices derived from the RH, PLM, and ROV being strongly correlated with each other, but not with FMD and its derivatives. The second major finding of this study is that leg vascular function, especially resistance artery function, is strongly related to V̇O_2_max, accounting for approximately 30% of the variance in V̇O_2_max not accounted for by known influencers, like body mass, sex, and BMI.

### 4.1 Are the various indices of vascular function interchangable with one another?

Multiple methods exist for quantifying a person's vascular function, yet it is unclear if these various methods are related to each other. Therefore, in the current study vascular function was measured in multiple ways (FMD, RH, PLM, and ROV) on a group of young, healthy adults. As illustrated in Figure 1 and further described in Table 1, lower limb vascular function assessed by the resistance artery tests RH, PLM, and ROV exhibits strong relationships with each other (*r* = 0.54–0.83, *p* < .05), supporting the notion that they reflect some of the same physiological processes (Limberg et al., 2020). This comes in agreement with data from Rossman, Groot, Garten, Witman, & Richardson (2016) and Walker et al. (2016) who observed significant correlations between PLM‐induced hyperemia and RH in various populations. However, as was the case for Rossman et al. (2016), vascular function assessed by FMD of the superficial femoral artery was not related to the other measurements of vascular function (e.g., PLM‐induced hyperemia) examined in the current study (Figure 1, Table 1).

The lack of relationship between FMD and the other variables should not be interpreted as evidence of superiority or inferiority of one test over another, but as an indication that these validated tests of vascular function capture different aspects of cardiovascular physiology. Indeed, principal components analysis, which consolidated the various indices of vascular function into two different factors, supports the idea that the results of the various tests capture two general aspects of vascular physiology. As illustrated in Table 1, Factor 1 is comprised exclusively of FMD and factors derived from the FMD test, which have been suggested to represent conduit artery function (Thijssen, Black, et al., 2011). Meanwhile, Factor 2 was comprised of the main indices derived from RH, PLM, and ROV, all of which have recently been referred to as tests of resistance artery or resistance vessel function (Limberg et al., 2020). Thus, the current data indicate that the tests of resistance artery function used in the current study are relatively interchangeable, but that tests reflecting conduit artery function should not be considered as surrogates for tests of resistance artery function, or vice versa.

### 4.2 Are conduit and/or resistance artery function related to V̇O_2_max?

The overarching aim of this study was to answer the question, “Is vascular function related to V̇O_2_max?” However, the data in Table 1 make it clear that one must clarify which aspect of vascular function is of interest when answering this question, since indices of conduit artery function and resistance artery function are not well correlated. As illustrated in Figure 2, resistance artery, but not conduit artery, function was strongly related to absolute V̇O_2_max, meaning that an individual with a large hyperemic response to the vascular tests would be likely to achieve a greater maximal rate of oxygen consumption and power output (e.g., GXTmax) during a graded exercise test. Meanwhile, mass‐specific V̇O_2_max was only related to resistance artery function assessed by PLM Peak Flow (*r* = 0.46, *p* = .03) and RH Total Flow (*r* = 0.41, *p* = .05), but not conduit artery function assessed by FMD (*r* = 0.20, *p* = .40). The strong relationship between resistance artery function and V̇O_2_max in these healthy young adults is consistent with previous studies that have reported relationships between V̇O_2_max and the hyperemic responses to RH (Robbins et al., 2011) and ROV (19) in various populations.

It makes sense that V̇O_2_max would be more related to resistance artery function than conduit artery function since V̇O_2_max is strongly influenced by maximum blood flow (Gifford et al., 2016; Levine, 2008) which is primarily controlled by the dilation and constriction of the myriad of resistance arteries (Dodd & Johnson, 1991; Joyner & Case y, 2015). Along these lines, our group recently reported that factors associated with resistance artery function (e.g., PLM Peak Flow and ROV Peak Flow) were very predictive of peak blood flow achieved during knee extension exercise, while FMD was not (Hanson et al., 2020). A large PLM, RH, or ROV response seems to be indicative of a limb with a network of resistance arteries that can accommodate high rates of blood flow, thereby facilitating a greater V̇O_2_max. Thus, interventions targeting resistance artery function may potentially have more impact on exercise tolerance in healthy adults than interventions seeking to improve conduit artery function. Future studies could potentially further examine the relationship between conduit artery function and V̇O_2_max by measuring conduit artery diameter during a V̇O_2_max test. Unfortunately, such precise diameter measurements are not currently possible during cycling exercise.

Contrary to our findings, previous research (Montero, 2015) has indicated that FMD is typically related to V̇O_2_max, most commonly the mass‐specific V̇O_2_max. The reason for the disagreement between findings may be due to measurement location. In contrast to most previous studies, which measured FMD in the arm, the current study compared vascular function, including FMD, assessed in the lower limb to cardiorespiratory fitness assessed during a predominantly lower‐limb exercise like cycling or running. Indeed, the aforementioned meta‐analysis (Montero, 2015) concluded “further studies are needed to elucidate the association of cardiorespiratory fitness with lower limb endothelial function.” As mentioned earlier, exercise‐induced adaptations to arterial structure and diameter appear to be of a greater magnitude in exercise‐trained muscles than in nontrained muscles (Rowley et al., 2012). It is possible that exercise‐induced adaptations in the diameter of the superficial femoral artery masked the relationship between FMD in the lower limb and V̇O_2_max. Thus, further investigation into the relationship between vascular function and V̇O_2_max, when controlling for potentially confounding variables, is warranted.

### 4.3 What other factors influence the indices of vascular function?

It is important to recognize that although these indices of vascular function are related to NO bioavailability and endothelial function (Casey & Joyner, 2011; Green, 2005; Mortensen et al., 2012), multiple other factors, besides endothelial function, can influence the results of these vascular function tests. As listed in Table 3, the measures of vascular function utilized in the current study are sensitive to several factors that should be considered when interpreting the results of a test. For example, in agreement with previous research (Anderson et al., 1995; Celermajer et al., 1992), FMD was negatively related to baseline artery diameter, such that individuals with a large diameter artery at baseline tend to exhibit a lower FMD. In the initial paper to link brachial artery FMD to coronary endothelial dysfunction (Anderson et al., 1995), the authors indicated that baseline brachial artery diameter was the strongest predictor of a decreased FMD, not the coronary endothelial dysfunction for which the paper is famous. With ~41% of the variation in FMD in the current sample being related to baseline diameter (i.e., *R* ^2^ = 0.41, *p* < .01), it is possible that the arterial enlargement associated with habitual exercise (Green et al., 2012) may have masked any potential relationship between FMD and V̇O_2_max in the current study.

As depicted in Table 3 tests of resistance artery function are strongly related with body mass and BMI, such that larger individuals with larger thighs tend to exhibit a greater RH Peak Flow, PLM Peak Flow, and ROV Peak Flow. It is not possible to conclude why this relationship exists from the current data, but it seems likely that larger limbs have a larger vascular network, which can accommodate greater flows. Whatever the mechanism, the influence of body mass on the measures of resistance artery function is not trivial and should be considered when interpreting these tests, especially when relating vascular function to V̇O_2_max, which is also strongly influenced by body mass (Proctor & Joyner, 1997).

Sex is also related to resistance artery function (Table 3), with males exhibiting a greater peak flow response to PLM. A similar tendency was also observed with ROV Peak Flow (*p* = .10). However, this sex difference in resistance artery function appears to be driven by differences in body mass between females and males (males were 25.35 ± 2.73 kg heavier than the females in this study, *p* < .01), since the sex differences in PLM Peak Flow disappeared when statistically removing variance in PLM Peak Flow accounted for by body mass (*p* = .98).

In addition to the factors mentioned above, previous research has revealed other factors that must be considered when performing and interpreting tests of vascular function. For example, the placement of the cuff proximal or distal to the site of measurement may impact the results of an FMD test (Doshi et al., 2001), the frequency of movement and the range of motion of PLM (Gifford et al., 2019), and the amount of work performed during ROV (Tschakovsky et al., 2004) have been shown to strongly impact the results. Therefore, these factors should be considered when exploring the relationship between vascular function and other variables, like V̇O_2_max.

### 4.4 Is vascular function related to V̇O_2_max when controlling for potentially confounding variables?

As described above, several factors, independent of the health of the vascular system, may impact the results of a vascular function test. Thus, it is possible that the underlying influences of variables, like artery diameter and body size, either mask potential relationships between vascular function and V̇O_2_max or potentially account for them. Partial correlations between the indices of vascular function and V̇O_2_max were performed to statistically remove variance accounted for potentially confounding variables. As described in Table 4, when statistically controlling for the variance in FMD related to baseline artery diameter, superficial femoral artery FMD does exhibit the weak relationship with mass‐specific V̇O_2_max (*r* = 0.45, *p* = .05) that has been indicated by studies measuring FMD in the arm (Montero, 2015). No such relationships were observed with absolute V̇O_2_max (*p* > .05). Thus, conduit artery function does appear to be weakly related to mass‐specific V̇O_2_max, but the relationship is obscured by variation in artery diameter.

While indices of resistance artery function are related to V̇O_2_max (Table 2), this relationship could potentially be completely dependent upon body mass, sex, and BMI, which are also strongly related to vascular function (Table 3) and V̇O_2_max (Table 2). Thus, the partial correlation between the indices of resistance artery function and V̇O_2_max was explored when simultaneously controlling for body mass, sex, and BMI. As described in Table 4, the relationship between resistance artery function and absolute V̇O_2_max persists, while the relationship between resistance artery function and mass‐specific V̇O_2_max is apparently strengthened when removing any variance in vascular function and V̇O_2_max related to body mass, sex, and BMI. Similarly, previous research indicated that PLM Peak Flow was related to peak exercise blood flow in a mass‐independent manner (Hanson et al., 2020). Thus, the relationship between resistance artery function and V̇O_2_max occurs independently and is not merely a product of sex, mass, or BMI.

As described by Wagner (Wagner, 2008), V̇O_2_max can be simultaneously influenced by the function of many systems, including the lungs, heart, arteries, skeletal muscle mass, and mitochondria. With so many factors influencing V̇O_2_max in healthy young adults that resistance artery function accounts for ~30% of the variance in V̇O_2_max not accounted for by body mass, sex, and BMI is quite notable. Factors that were not measured in the current study, like maximal cardiac output, mitochondrial density, and muscle oxygen diffusion are likely to account for some of the remaining variance (Gifford et al., 2016; Wagner, 2008). Since V̇O_2_max is limited by different factors in different populations (Gifford et al., 2016; Wagner, 2008), the amount of variance in V̇O_2_max accounted for by resistance artery function likely differ in other populations.

### 4.5 Clinical relevance

Since V̇O_2_max strongly influences exercise performance and is also a strong predictor of cardiovascular risk (Poole et al., 2020), there is great interest in identifying what limits or reduces an individual's V̇O_2_max (Wagner, 2008) so that appropriate steps may be taken to improve it. With resistance artery function being related to both maximum exercise blood flow (Hanson et al., 2020) and V̇O_2_max (Table 2), noninvasive assessments, like passive‐leg movement (PLM)‐induced hyperemia, may conceivably be used to easily determine the likelihood that impairments in muscle resistance artery function and leg blood flow impair a person's V̇O_2_max. Since the PLM technique occurs while the subject is in a completely rested state, this could be particularly useful in scenarios in which direct assessment of exercise blood flow may not be possible or practical.

Given the strong relationship between resistance artery function and V̇O_2_max, vascular function data collected at rest could potentially be used to predict V̇O_2_max. For example, stepwise linear regression revealed that absolute V̇O_2_max (expressed in ml·min^−1^) could be predicted (*R* ^2^ = 0.83, *p* < .01, *n* = 22) when considering the peak flow response to PLM (expressed in ml·min^−1^), body mass (expressed in kg), and BMI (expressed in kg·m^−2^):

$$\begin{array}{cc} \text{Absolute}\;\dot{V}O_{2}\max & =970.82+55.83\left(\text{Body Mass}\right)\\ & \;+0.68\left(\text{PLM Peak Flow}\right)‐121.75\left(\text{BMI}\right). \end{array}$$

Clearly, these data are very preliminary, and a much larger, more heterogeneous sample is needed before a prediction equation may be validated and standardized, but the prospect of accurately predicting V̇O_2_max without breaking a sweat is enticing.

### 4.6 Conclusions

This study supports the notion that noninvasive indices of vascular function generally reflect two different aspects of vascular function: conduit artery function (e.g., FMD) and resistance artery function (e.g., RH Peak Flow, PLM Peak Flow, and ROV Peak Flow). Importantly, the results of the tests within each aspect of vascular function (i.e., conduit or resistance artery function) relate well to one another, such that inferences about one test may be made based on the results of another. While only a weak relationship between conduit artery function (*e.g*. FMD) and V̇O_2_max is observed when accounting for baseline artery diameter, resistance artery function, assessed by multiple different tests, is consistently and independently related to V̇O_2_max. While FMD has been related to various aspects of cardiovascular health (Broxterman et al., 2019), it is the function of the resistance arteries, not the conduit arteries, that is tightly related to exercise capacity and physical function. Thus, vascular interventions, like exercise training (Montero, Walther, Diaz‐Cañestro, Pyke, & Padilla, 2015), seeking to improve exercise capacity should target resistance artery function, as represented by factors like peak flow during PLM, RH, or ROV.

## AUTHOR CONTRIBUTIONS

JG: Designed and performed the study, analyzed the data, and wrote the manuscript. BH: Designed and performed the study and wrote the manuscript. MP: Performed the study, analyzed the data, and approved the final manuscript. TW: Performed the study, analyzed the data, and approved the final manuscript. GG: Performed the study, analyzed the data, and approved the final manuscript. JK: Performed the study, analyzed the data, and approved the final manuscript. MH: Performed the study, analyzed the data, and approved the final manuscript.
